# Integrated maternity care: A concept analysis

**DOI:** 10.1371/journal.pone.0306979

**Published:** 2024-08-01

**Authors:** Evelien Cellissen, Ruben van Zelm, Marijke Hendrix, Hajo I. J. Wildschut, Marianne Nieuwenhuijze

**Affiliations:** 1 Department of Midwifery Education and Studies, Research Centre for Midwifery Science, Zuyd University, Maastricht, The Netherlands; 2 University of Applied Sciences, Utrecht, The Netherlands; 3 Wildschut Consultancy, Utrecht, The Netherlands; 4 Care and Public Health Research Institute (CAPHRI), Maastricht University, Maastricht, The Netherlands; National Institute of Public Health, MEXICO

## Abstract

**Introduction:**

Integrated maternity care is strongly promoted in the Netherlands. However, the term ‘integrated’ and its practical meaning is understood differently by professionals and policy makers. This lack of clarity is also visible in other countries and hinders implementation. In this study, we will examine how the concept of ‘integrated maternity care’ and its defining attributes are presented in the international literature.

**Methods:**

This study aims to provide a definition and deeper understanding of the concept of integrated maternity care by conducting a concept analysis using Morse’s method. We performed a systematic search using Embase and Ebscohost (CINAHL, PsychINFO, SocINDEX, MEDLINE) including records that described integrated maternity care from on organizational perspective. Through a qualitative analysis of the selected research and non-research records, we identified defining attributes, boundaries, antecedents, and consequences of the concept. Subsequently, we constructed a definition of the concept based on the findings.

**Results:**

We included 36 records on integrated maternity care in the period from 1978 to 2022. Our search included 21 research and 15 non-research records (e.g. guidelines and policy records). Only half of these had a definition of integrated maternity care. Over time, the definition became more specific. Our concept analysis resulted in three defining attributes of integrated maternity care: collaboration, organizing collaboration and woman-centeredness. We identified role clarity, a culture of collaboration, and clear and timely communication as antecedents of integrated maternity care. A number of consequences were found: continuity of care, improved outcomes, and efficiency. All consequences were described as expected effects of integrated maternity care and not based on evidence.

**Conclusion:**

We propose the following definition: ‘Integrated maternity care is woman-centred care provided by (maternity) care professionals collaborating together within and across different levels of healthcare with a specific focus on organizing seamless care.’ Addressing the antecedents is important for the successful implementation of integrated maternity care.

## Introduction

In the past two decades, integrated care has become an important concept in healthcare reforms worldwide [1–4]. According to the World Health Organisation, integration of care is a way to improve access, quality, user satisfaction, and efficiency of care [4]. This is achieved by creating coherence and synergy between various parts of the healthcare system. The focus of integrated healthcare was originally on complex and multi-problem patients because these patients are most affected by poor access, lack of continuity, and fragmentation in healthcare settings [5]. Over time, integrated care also became increasingly important for general healthcare as a way to sustain healthcare systems to achieve equity, effectiveness, and efficiency of care [5,6].

In the Netherlands, an emphasis on integrated maternity care is seen over the past years [7–9]. Much attention is paid to improving interprofessional collaboration among healthcare professionals involved in maternity care, such as obstetricians, midwives, and nurses. This was evoked by the report of the EURO-PERISTAT project in 2008 [10]. This report indicated that the perinatal mortality rate in the Netherlands was relatively high compared to other European countries. A national Dutch Steering Group suggested that the organization of the Dutch maternity care system might be one of the possible causes of this relatively high mortality rate [11]. Maternity care in the Netherlands includes preconception, prenatal, natal, and postnatal care. Women’s care is based on the assessment of the individual risk of each woman where women with a low-risk pregnancy are cared for by community midwives while women at intermediate or high risk are referred to secondary or tertiary healthcare professionals (obstetricians or hospital-based midwives) [12]. This division in community and hospital-based healthcare might cause suboptimal alignment of care according to a report of the Dutch Steering Group [11].

In a response to this Steering Group report, the Integrated Maternity Care Standard was developed in 2016 [7]. This care standard sets the norm for the organization of preconception, prenatal, natal, and postnatal care in an integrated way for each pregnant woman. The Integrated Maternity Care Standard addresses the organizational requirements for a safe, effective, and woman-centered maternity care system, emphasizing closer regional collaboration between maternity care professionals in community- and hospital-based healthcare.

Even though integrated maternity care is strongly promoted in the Netherlands, the term ‘integrated’ and its practical meaning are understood differently by professionals and organizations involved in maternity care [13]. Turning to international literature, a similar inconsistency was seen where the term ‘integrated’ was widely used in different contexts and with different meanings [3,6,14]. This indicates a lack of clarity of what is actually meant by the concept of ‘integrated care’ and specifically ‘integrated maternity care’. The concept seems strongly shaped by the perspectives and expectations of the different actors. Despite the popularity of the concept of integrated maternity care and its extensive use, the concept remains implicit and multi-interpretable. “The lack of clarity of the concept of integrated maternity care is a barrier to further development of integrated maternity care in practice. Without a clear understanding of this concept, care professionals may interpret and apply this concept differently, leading to inconsistencies in practices [13]. In addition, the lack of clarity of the concept of integrated maternity care also poses barriers to conducting research on its effectiveness, preventing policymakers from basing their recommendations on evidence. Thus, a shared and unified definition of the concept of integrated maternity care is necessary to foster better understanding and development in practice, and ensure the future progress of integrated maternity care.” [3,4,15].

Several literature reviews have been published on integrated maternity care, but these reviews focus either on specific elements of maternity care like pregnancy or on its implementation [14,16–21]. Additionally, these authors use various definitions of integrated maternity care. To the best of our knowledge, no systematic exploration of the concept of integrated maternity care has been undertaken and published in international literature. The research question in this study is: ’What is the definition of integrated maternity care and how is the concept of integrated maternity care described, including defining attributes, boundaries, antecedents, and consequences? This study aims to provide a definition and deeper understanding of the concept of integrated maternity care by conducting a concept analysis.

## Methods

We performed a concept analysis to clarify and delineate the definition of integrated maternity care in the context of organizing integrated maternity care.

**Fig 1:**
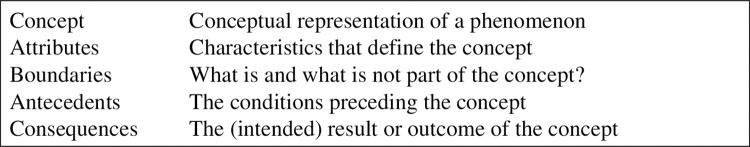
Relevant terms in Morse’s method of concept analysis [22].

Concepts are the basis for theory and research on a specific topic. According to Morse [22], a concept has to be mature to be able to use it in research, to avoid confusion and misunderstanding. A mature concept is well-defined, and has clearly described attributes, delineated boundaries, antecedents, and consequences. The fact that currently, the concept of integrated maternity care is multi-interpretable, illustrates that it is still an immature concept. Therefore, we chose Morse’s method [22] for analysing defining attributes, boundaries, antecedents, and consequences to achieve a better understanding and a more conclusive definition of the concept. Fig 1 explains the terminology used in the concept analysis of this article.

### Process of concept analysis

**Fig 2:**
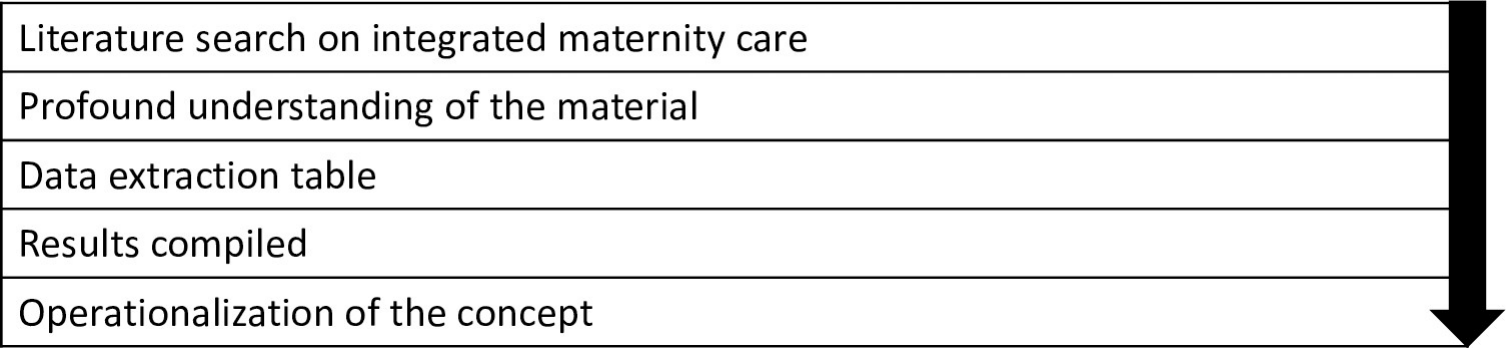
Process of concept analysis according to Morse [22].

Fig 2 presents the steps we followed to conduct our concept analysis.

#### Step 1: Literature search

**Fig 3:**
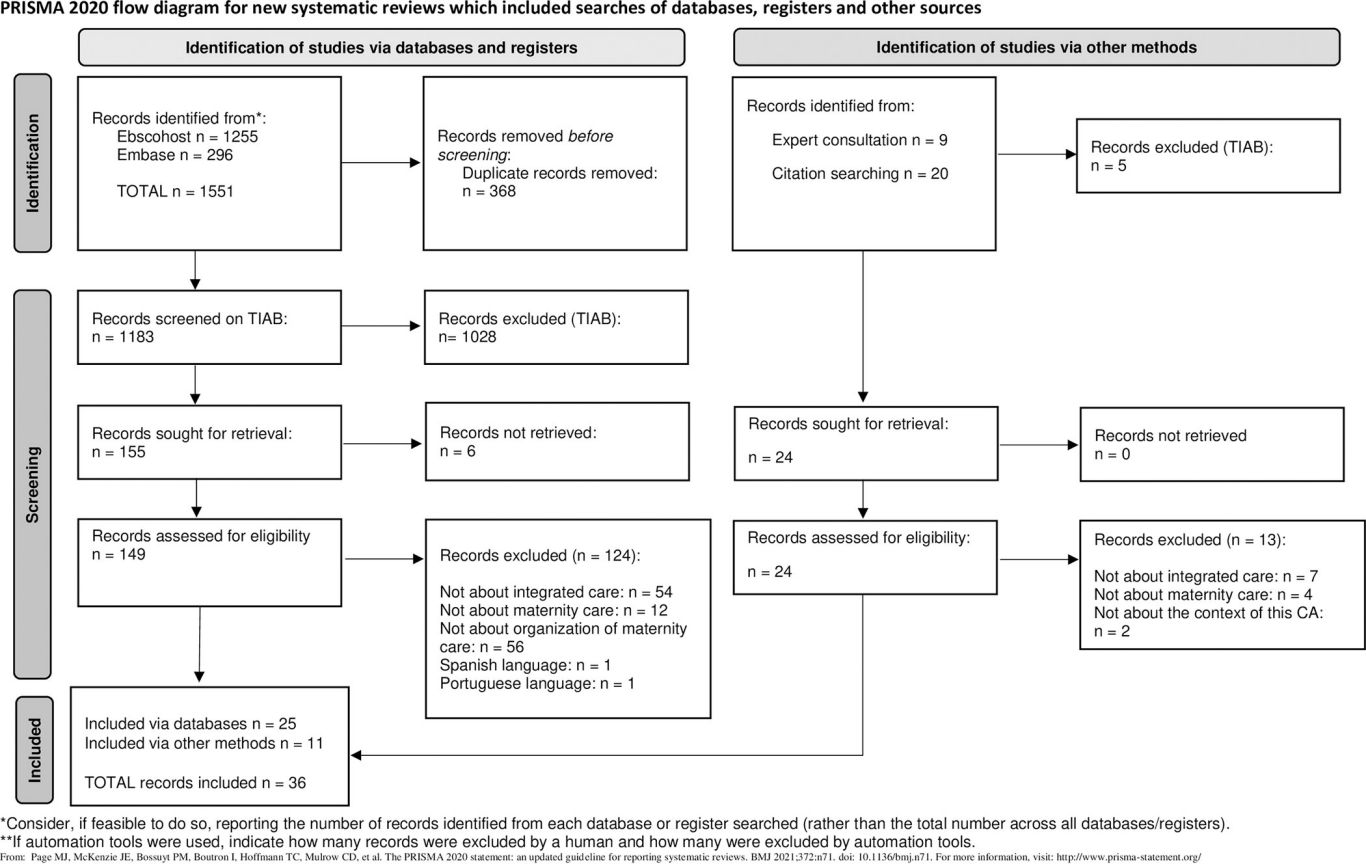
Flowchart of the systematic search.

We performed a literature search in April 2020, with an update in December 2021, and May 2023. We searched Embase and Ebscohost, including the databases CINAHL, PsychINFO, SocINDEX, and MEDLINE. The key concepts for the searches were ‘integrated care’ AND ‘maternity care’, and their synonyms (Table 1). We included records providing a definition or framework for organization of integrated care in the context of maternity care. We excluded records on related but different concepts (e.g. team-based care), records focused on integrating content of care from other healthcare domains into maternity care (e.g. integrating mental healthcare or HIV care into prenatal visits), and records on maternity care for specific populations (e.g. diabetes patients or preterm neonates) as these did not fit within the scope of our study (Table 2). We included only English and Dutch records and applied no publication date limitations. We included records where the search term(s) appeared in the title, abstract, and/or full text, ensuring a comprehensive approach to gathering relevant literature. As all literature is seen as data in a concept analysis, we included both records from peer reviewed scientific journals (including original research, reviews, discussion records, and congress abstracts) and other non-scientific documents (e.g. guidelines and policy records) [23]. Morse’s method does not require quality assessment of the individual records and for that reason we did not perform this [22,24].We followed the PRISMA (Preferred Recording Items for Systematic reviews and Meta-analysis) statement for conducting and recording the in- and exclusion process (Fig 3) with the exception of the appraisal of the literature as this is not a required step in Morse’s method [22,24,25].

**Table 1:** Search strategy.

Key concepts	Integrated care		Maternity care
**Synonyms**	“integrat* care” OR “integrat* health system” OR ”integrat* model” OR “integrat* health service*” OR “integrat* system” OR “shared care”	A N D	childbirth OR birth OR obstetr* OR maternity OR midwi* OR gynecolo* OR gynaecolo* OR reproducti* OR pregnan*

**Table 2:** In- and exclusion criteria.

***Inclusion criteria*** Providing a definition or framework for organization of integrated care, in the context of maternity care English and Dutch language No publication date limitations Scientific as well as non-scientific documents
***Exclusion criteria*** Other concepts about organizing maternity care than integrated maternity care (e.g. team-based care) Integrating specific content of care from other domains into maternity care Maternity care for specific populations

The first author performed the selection by screening titles and abstracts, monitored by the last author. Titles and abstracts without agreement between first and last author were included in the full-text screening. We obtained full-text records from the selected titles and reviewed these for relevance and applicability to the concept of the present study and meeting our inclusion criteria. Records that did not meet the inclusion criteria, were excluded. To complete the search, we hand searched the reference lists of included records and asked an expert on integrated maternity care for additional records.

#### Step 2: Studying the material

After selecting the records, we studied their content to gain an in-depth understanding of the concept integrated maternity care. We specifically studied possible attributes, boundaries, antecedents, and consequences as these are the relevant terms in Morse’s method of concept analysis (Fig 1).

#### Step 3: Data extraction table

All relevant information from these records was incorporated in a purpose-built data-extraction table containing the terms pointed out in Fig 1. The data-extraction table summarizes the definitions given in the records and the identified concepts divided into defining attributes, boundaries, antecedents, and consequences of integrated maternity care. This data-extraction table was used to analyse the data by the first and last author.

#### Step 4: Results compiled

The data-extraction table provided overview and allowed for critical content analyses of the records. The content was evaluated for clarity and different meanings and interpretations of the concept, which was discussed in several sessions with all authors.

#### Step 5: Operationalization of the concept

These author-discussions ultimately allowed us to operationalize the concept of integrated maternity care. Therefore we translated the different definitions into a uniform definition of integrated maternity care. Additionally, we described the concept by clarifying the attributes, boundaries, antecedents and consequences. Although we did not conduct a quality assessment of the records, we did identify the type of record (e.g. research record, policy record) and mentioned this in the results (Table 3).

**Table 3:** Overview of the included records.

Author and year	Title	Country	Type of record	Research method
Adeniyi et al, 2021 [26]	A Qualitative Study of Health Care Providers’ Views on Integrating Oral Health into Prenatal Care.	Canada	Research	Qualitative (interviews)
Afrizal et al, 2020 [27]	Evaluation of integrated antenatal care implementation in primary health care: study from an urban area in Indonesia.	Subsahara & Asia	Research	Mixed method (qualitative, interviews and quantitative, patient record data)
Angood, 2010 [28]	Blueprint for Action. Steps Toward a High-Quality, High-Value Maternity Care System.	USA	Conference summary	Not applicable–policy based
Barnea et al, 2021 [19]	From fragmented levels of care to integrated health care: Framework toward improved maternal and newborn health.	UK	Research	Literature review
Boesveld et al, 2017 [29]	Typology of birth centres in the Netherlands using the Rainbow model of integrated care: results of the Dutch Birth Centre Study.	The Netherlands	Research	Mixed method (qualitative, interviews and quantitative, surveys)
Cellissen et al. 2018 [30]	Integrating care by payment reform A workshop on the implementing of bundled payments for birth care in the Netherland.	The Netherlands	Conference abstract	Not applicable–experience based
College Perinatale Zorg (Perinatal Care Board), 2014 [31]	Samenwerking in de geboortezorg: positieve ontwikkelingen, knelpunten en oplossingen. (Collaboration in maternity care: positive developments, barriers and solutions)	The Netherlands	Policy	Not applicable
College Perinatale Zorg (Perinatal Care Board), 2016; 2020 [7]	Zorgstandaard integrale geboortezorg (Integrated maternity care standard)	The Netherlands	Guideline	Not applicable
Coupland et al, 2019 [17]	Developing a model of care for Substance Use in Pregnancy and Parenting Services in Sydney, Australia.	Australia	Conference abstract	Mixed method (literature review and qualitative, interviews)
Franx & Steegers, 2019 [32]	Verloskunde in transitie. (Maternity care in transition.)	The Netherlands	Opinion	Descriptive literature based on guidelines.
Hall et al, 2006 [33]	A qualitative study of an integrated maternity, drugs and social care service for drug-using women.	Scotland	Research	Qualitative (interviews)
De Jongh et al, 2016 [14]	Integration of antenatal care services with health programmes in low- and middle-income countries: systematic review.	(review)	Research	Systematic review
Kennedy et al, 2020 [34]	The role of midwifery and other international insights for maternity care in the United States: An analysis of four countries	The Netherlands, USA, Canada, Autralia	Research	Mixed method (qualitative, interviews and quantitative, national data on women)
Mattocks, 2019 [35]	Understanding Maternity Care Coordination for Women Veterans Using an Integrated Care Model Approach.	USA	Research	Quantitative (surveys)
Mayer & Bick, 2020 [20]	To what extent does UK and Irish maternity policy and guidance address integration of services to meet needs of women with comorbidity? A policy document review.	UK/Ireland	Research	Policy document review
McFarland, 2020 [36]	The experiences of midwives in integrated maternity care: A qualitative metasynthesis	USA	Research	Qualitative meta-synthesis
Nicolson, 2005 [37]	Shared maternity care: all care-not enough responsibility? An audit of patient care communications pre- and post- a multi-faceted intervention.	Australia	Research	Quantitative (patient record data)
NVOG, 2018 [38]	Integrale geboortezorg werpt eerste vruchten af. (Integrated maternity care is paying off first.)	The Netherlands	Policy	Not applicable
O’connor, 2017 [39]	The universal, collaborative and dynamic model of specialist and advanced nursing and midwifery practice: A way forward?	Ireland	Research	Qualitative (interviews)
O’Keeffe, 1997 [40]	Managed obstetrical care.	USA	Policy	Not applicable
Perdok et al, 2014 [41]	Opinions of maternity care professionals about integration of care during labor for “moderate risk” indications: a Delphi study in the Netherlands.	The Netherlands	Research	Qualitative (Delphi study)
Perdok et al, 2016 [42]	Opinions of maternity care professionals and other stakeholders about integration of maternity care: a qualitative study in the Netherlands.	The Netherlands	Research	Qualitative (interviews and focus groups)
Pieters et al, 2018 [16]	Improving inter-organizational care-cure designs: specialization versus integration.	The Netherlands	Research	Mixed method (literature review; qualitative, interviews; quantitative, surveys and data)
Posthumus et al, 2013 [18]	Bridging between professionals in perinatal care: towards shared care in the Netherlands	The Netherlands	Research	Descriptive literature review
Prins et al, 2013 [43]	Advantages of midwife-led continuity model of care.	The Netherlands	Opinion	Not applicable–opinion based
Rogers, 2003 [44]	Podium: doctors speak out. Sustainability and collaboration in maternity care in Canada: dreams and obstacles.	Canada	Opinion	Not applicable–opinion based
Romijn et al, 2017 [45]	Interprofessional collaboration among care professionals in obstetrical care: are perceptions aligned?	The Netherlands	Research	Quantitative (surveys)
Schlaefer, 2017 [46]	Bridging Health and Social Care—An Innovative Framework.	Israel	Conference abstract	Not applicable–experience based
Stuurgroep (Steering group), 2009 [11]	Een goed begin: veilige zorg rond zwangerschap en geboorte. (A good start: safe maternity care)	The Netherlands	Policy	Not applicable
Thomas et al, 1987 [47]	Evaluation of an integrated community antenatal clinic.	UK	Research	Quantitative (quasi-experiment–control group)
The Multidisciplinary Collaborative Primary Maternity Care Project, 2006 [48]	Guidelines for development of a multidisciplinary collaborative primary maternity care model	Canada	Policy	Not applicable–guideline based
Vedam et al, 2018 [49]	Mapping integration of midwives across the United States: Impact on access, equity, and outcomes	USA	Research	Qualitative (Delphi study)
De Vries, 2018 [50]	How (not) to create value based integrated maternity care: Lessons from the Netherlands	The Netherlands	Conference abstract	Not applicable–opinion based
Van der Werf et al, 2022 [21]	Integrated Maternal Care Strategies in Low- and Middle-Income Countries: A Systematic Review	Review	Research	Systematic review
Wildschut & Boesveld, 2018 [9]	Integrale geboortezorg. Samen bevalt goed. (Integrated maternity care.)	The Netherlands	Textbook	Not applicable
Zander, 1978 [51]	Integration of general-practitioner and specialist antenatal care.	USA	Research	Quantitative

## Results

The database literature search yielded 1,551 records, leaving 1,183 records after removing duplicates. After screening titles and abstracts, we excluded 1,028 records, leaving 155 potentially relevant records for full-text review. Six records were not included because we could not retrieve the full-text. Of the 149 full-text records, we excluded 124 records. The main reasons for exclusion were: the record was not about integrated care (n = 54), not about maternity care (n = 12), not about organization of integrated care (n = 56) or in Spanish/Portugese language (n = 2). As a result, 25 records met the inclusion criteria and were included for further analysis. An additional 29 records were found through hand searching and consulting an expert on integrated maternity care, from which 11 met the inclusion criteria. A total of 36 records was included in this concept analysis, published between 1978 and 2022 (Fig 3). An overview of the included records is available in Table 3. We included 20 records from European countries, 6 from Northern America, 2 from Australia, 3 from Canada, 2 from other countries and 3 studies conducted in multiple countries. We included different types of records: 21 reporting research and 15 non-research records like conference abstracts, policy records or guidelines.

### Definition integrated maternity care

**Fig 4:**
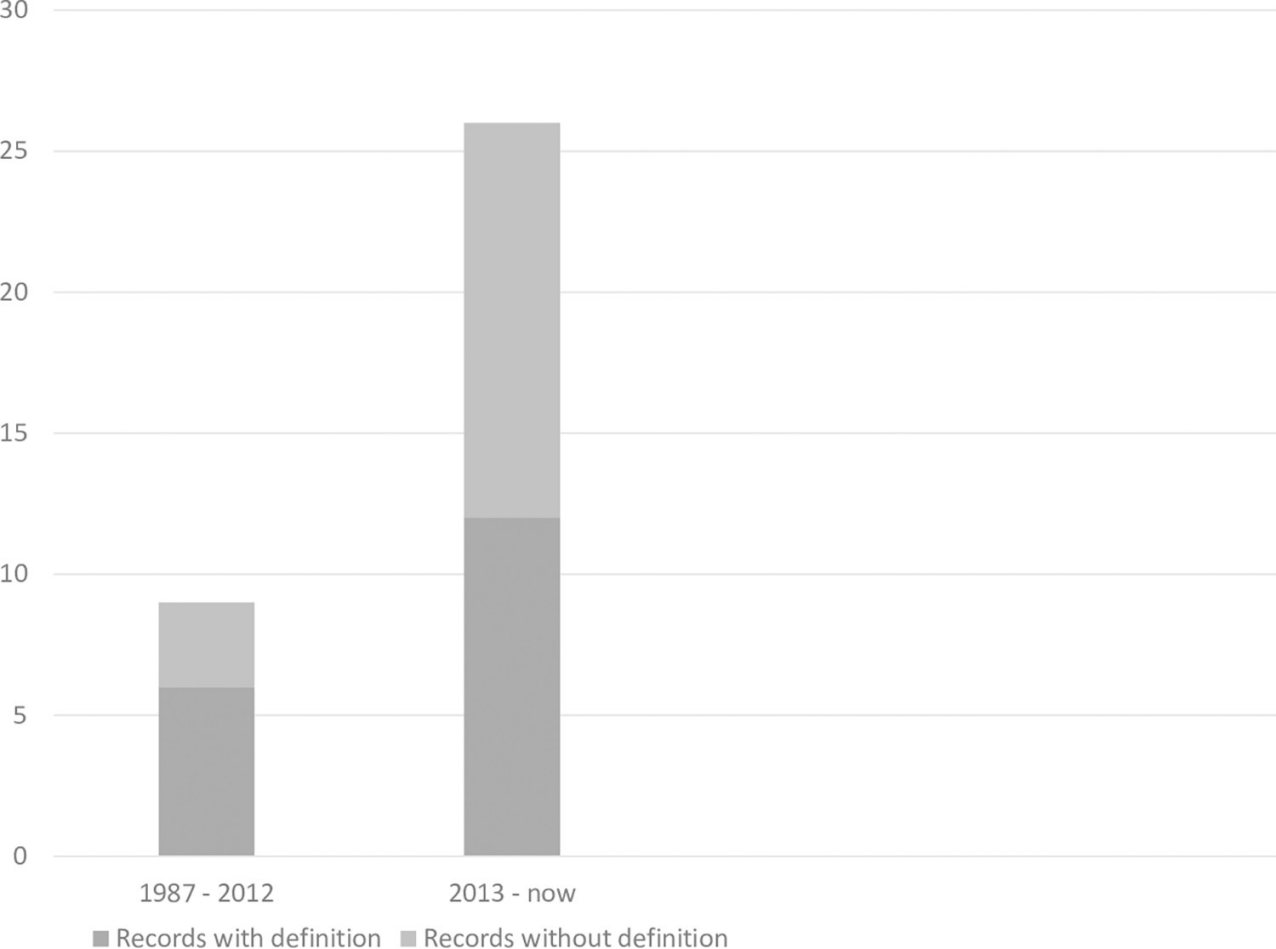
Development definition integrated maternity care over time.

Of the 36 records, 18 had a definition of integrated maternity care [7,9,11,17,32,49]. Regarding the definition, we noticed a shift over time (Fig 4). The first record we found with a definition on integrated maternity care, dated from 1987 [47]. In a period of 25 years, from 1987 till 2012, six out of nine records on integrated maternity care provided a definition [11,37,40,44,47,48]. In the last decade, from 2012 till 2022, we found an increasing number of records, where 12 records out of 27 gave a definition on integrated maternity care [7,9,17,26,29,31,32,35,36,41,42,49]. Only five records created their own definition on integrated maternity care [9,26,29,41,49], where all others referred back to the definitions on integrated care used in other records.

Over time, the definitions of integrated maternity care became more specific. Until 2009, integrated maternity care was generally defined as a model in which there is collaboration between midwives and obstetricians or between primary and secondary maternity care [37,40,44,47,48]. Terms like responsibility and continuity of care were added to the definitions since 2009 [7,11,31,41]. From 2016, the woman’s perspective was added to the definitions [9,29,35,42] for example women’s satisfaction [35] and mentioning the pregnant woman explicitly as a collaboration partner within integrated maternity care [9,35]. Finally, from 2019, an organizational context was added to definitions [21,26,32,36]. The basic referral to collaboration between different maternity care professionals was included in all definitions over time.

### Defining attributes of integrated maternity care

**Fig 5:**
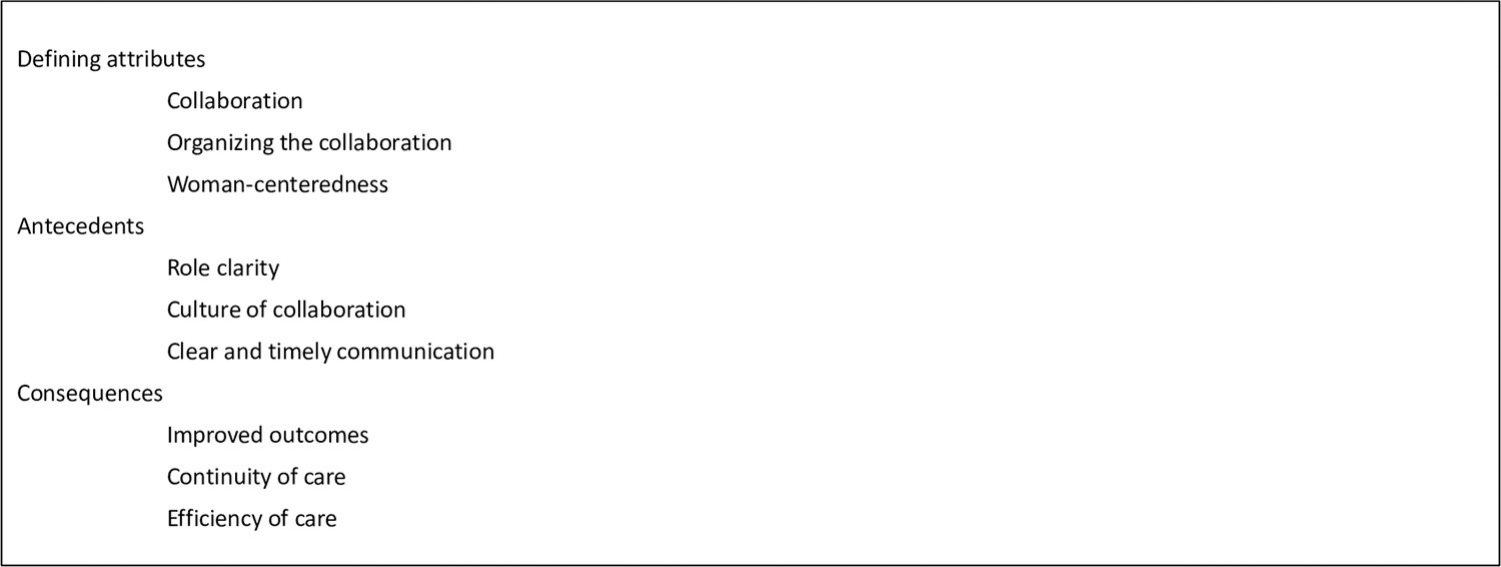
Defining attributes, antecedents, and consequences of integrated maternity care.

Three distinctive attributes of integrated maternity care appeared from the literature, clarifying the understanding of the concept (Fig 5):

1. Collaboration
2. Organizing the collaboration
3. Woman-centeredness

#### Collaboration

Collaboration was mentioned in all included records and generally defined as being about interaction between different actors. This interaction was defined on different levels of healthcare: system, sector, organization, and professional. System level is about integrating different health systems, sector level is about primary and secondary care, organization level is about different organizations and professional level is about the different professionals involved. Some records suggested that integrated care was about collaboration between different systems. Collaboration at system level was described between the social system and health system [9,33,40,46]. Next to this, at sector level, the interaction between different sectors was mentioned as collaboration between primary and secondary care [7,9,11,17,19–21,27,31,32,34,37,38,41,42,45–47,49,51]. At organization level, various collaborations between organizations, each with their own governance code, policy or location was mentioned, for example, collaboration between community-midwife practices and maternity wards in hospitals [7,11,16–18,20,21,26,27,29–31,33,35,37,38,41,42,45–47,49,51]. Finally, at professional level, collaboration between a diversity in the types of professionals involved in integrated maternity care was mentioned. Almost all records suggested collaboration between (community) midwives and obstetricians as being part of integrated maternity care. In addition, some included nurses [7,9,31,34,36,38–41,44,45], related healthcare professionals like paediatricians or physician assistants [7,9,11,18,19,27,31,35,36,39,40,46], and/or general practitioners [7,9,11,18,31,33–35,37,42,47,51].

#### Organizing the collaboration

Organizing the collaboration within and across different levels of healthcare is mentioned in almost all records as an important attribute of integrated maternity care [7,9,11,14,16–18,20,21,26–28,30–36,38–40,42,46–48]. The way in which integrated maternity care was actually organized, is diverse. We found no uniform organization profile but a range from informal networks to merged organizations [7,11,16,21,26,28,31,32,34,38–40]. However, the need to coordinate the collaboration was mentioned in many records, both research and non-research [7,9,11,14,17–21,26–28,31,34–36,38,40,46,48]. This coordinated collaboration was named differently in the included records. Terms like ‘network’ [11,21,26,31,34,38,40], ‘collaborative model’ [16], ‘maternity care collaboration’ [7,25], ‘partnership’ [39] or ‘system of care collaboration’ [28] were used in different record types.

It was also diverse how this coordination should be fulfilled. Some authors, suggested a role for a leader or coordinator or at least someone who facilitates the professionals in working together and reinforce collaborative behavior at the level of organizing care [17,18,27,44]. Other authors, in research as well as non-research records, suggested that a coordinator was essential to organize the care at professional level between the woman and the professional(s) [11,17,18,20,21,27,35,40,46]. Next to coordinating the care, an adequate referral system was mentioned in some records [14,20,34,39]. This should allow for pregnant women to be referred in time to the appropriate professional within the network if necessary, ensuring that the involved actors collaborate adequately. Finally, some non-research records suggested a change in the way the health care system was financed as part of the organization of integrated maternity care [9,30,38,40,42]. These options for financial change were suggested as general recommendations and were not supported by research.

#### Woman-centeredness

Integrated maternity care is about providing woman-centered care. Angood et al (2009) were the first authors in the selected records, who mentioned woman-centeredness as an attribute to integrated maternity care. After that, the role of women increased in records on integrated maternity care [17,18,21,29,35,41,42] and since 2016, the woman’s perspective became an explicit part of the definition of integrated maternity care [9,29,35,42,49]. Woman-centeredness was not clearly defined in most included records. Perdok et al. (2016) mentioned that input from the woman in choices around the management of care and decision-making was essential to achieve this [42]. Others described woman-centeredness in integrated maternity care as care that was tailored to the woman’s needs and preferences; and based on shared responsibility between a woman and her caregiver for optimizing her health and that of the baby [7,9,35].

### Boundaries of integrated maternity care

Several records used the concept integrated maternity care but did not include all the above attributes: collaboration, organizing the collaboration and woman-centeredness. Collaboration was mentioned in all records. Of the 36 records, 9 described collaboration on system, sector, organization, or professional level but did not mention the way to organize this collaboration [29,37,41,43–45,49–51]. These records lack the defining attribute ‘organizing the collaboration’. Of all 36 records, only 13 included woman-centeredness as a defining attribute for integrated maternity care [7,9,11,17,18,21,28,31,32,35,41,42,46]. We conclude that only eleven, mostly non-research, records used all defining attributes of the concept of integrated maternity [7,9,11,17,18,21,28,31,32,35,46].

### Antecedents of integrated maternity care

In this concept analysis, we identified role clarity, culture of collaboration, and clear and timely communication as antecedents of integrated maternity care (Fig 5).

#### Role clarity

Role clarity of the involved professionals came forward from the included records as an important antecedent for integrated maternity care. When roles are clearly defined, it enables professionals to perform the role they are best suited. In many of the research records, role clarity was described, in terms as ‘specific expertise’, ‘competencies and skills’, ‘responsibilities’, and ‘strengths and limitations’ of the professionals [9,16–18,21,26,27,34,41,42,44]. Most of these terms were not further explained. However, different views were expressed on dealing with responsibilities. Where some records suggested that professionals should work with equal or shared responsibilities [7,11,18,38,41], others suggested a strict distinction between tasks and each professional being autonomously responsible [41,42]. None of the records involved research on what interpretation of responsibilities works best to establish integrated maternity care.

#### Culture of collaboration

A culture of collaboration is another antecedent to integrated maternity care. In the included records, different elements were used to describe this culture of collaboration. Mostly mentioned were ‘sharing a vision and values about maternity care’ [9,18,27,29,41,42,44,46,48], and ‘mutual trust and respect’ [9,11,17,18,34,38,44] between all professionals involved. Several research records suggested that implementing integrated maternity care was easier when the involved professionals, whether or not working in the same organization, shared their vision on values in maternity care, and had mutual trust and respect [9,11,17,18,27,29,34,38,41,42,44,46,48]. One of the important requirements in this, mentioned in research records, seemed exploring different perspectives, a willingness to discuss these differences [18,27,36,41,44–46], and the willingness to give critical feedback [17,18,27,44]. A few authors suggested in their research report that a way to achieve a more collaborative culture, adequate staff resources were important to build trust, lead, guide, and support the involved professionals [17,18,27,44]. Other authors mentioned the importance of inter-professional education and training contributing to knowing and respecting each other’s skills and thereby contributing to achieving a collaborative culture [9,18,19,21,26,34,40,41].

#### Clear and timely communication

Clear and timely communication between involved professionals was also described as an important condition for providing integrated maternity care. This antecedent was based on two elements: clear agreements and medical information sharing between the professionals. The need to make clear agreements between organizations as well as between professionals about the content of the medical and obstetrical care was suggested in several, mostly research, records [9,17–20,26,27,34,40,41,47,48]. Terms used for these agreements were ‘interprofessional guidelines’ [26,27,40,41], ‘multidisciplinary care pathways’ [17–20,34] or ‘working arrangements’ [9,11,47]. The second element necessary to provide clear and timely communication in integrated maternity care was timely sharing medical information about the woman with all the professionals involved [7,9,11,21,26,27,31,37,38,45–47,51]. Four of these records specifically mentioned a shared IT system as conditional to share this medical information [7,18,27,46]. Three records also suggested meetings between involved professionals to discuss the care to be provided and thereby coordinate and share medical information [7,18,45]. Although this was mentioned in research records, these meetings were not considered as study outcomes but were suggested as potential contributors to clear communication.

### Consequences of integrated maternity care

Three consequences of integrated maternity care were mentioned in the records: improved outcomes, continuity of care, and improvement in efficiency of maternity care *(*Fig 5).

#### Improved outcomes

Of the three mentioned consequences of integrated maternity care, improved outcomes was mentioned most in the records. These outcomes included perinatal outcomes and women’s experiences with maternity care. The outcomes were mostly based on expected outcomes when records were suggesting models or definitions of integrated maternity care. The outcomes were described in general terms such as improved perinatal outcomes [7,9,11,20,30,31,36,38,50]. There were some records who based their results on research, here outcomes were further specified [14,21,33,34,36,47,49,51]. For example, Thomas et al (1987) reported fewer women suffering from hypertension when comparing integrated antenatal care with diverse professionals on one location with shared care offered by midwives and obstetricians in different locations [47]. The other records did not report specific outcomes or any statistical information on increasing or decreasing perinatal outcomes. According to women’s experiences with maternity care, only Thomas et al. (1987) and De Jongh et al. (2016) reported increased women’s satisfaction (e.g. shorter waiting time) with integrated maternity care based on research [14,47]. A few other records mentioned an expected improvement in women’s experiences with maternity care when integrating maternity care, which was not based on research results [48,50].

#### Continuity of care

Continuity of care was also mentioned as one of the consequences of integrated maternity care. It was described as the importance of seamless collaboration between actors involved in maternity care within and across organizations, sectors or systems [9,14,18,20,21,31,33,39,41–43,47,51]. The aim of continuity of care was to prevent segmentation in the care of women. When providing integrated maternity care, continuity of care was improved according to five research records [14,21,33,47,51]. Three of these records based their results on reported patient experiences with integrated care [33,47,51]. The context of care and the definitions of integrated maternity care were different in these records.

#### Efficiency of care

Improvement in efficiency of care was pointed out in a few records as an expected consequence of integrated maternity care [9,26,34,40,47]. Most of these records described expected improvement in efficiency for example based on avoiding duplication of consultations or visits by using a care pathway [9,34]. We found no research results in our data about improving efficiency when providing integrated maternity care.

## Discussion

The research question in this study is: ’What is the definition of integrated maternity care and how is the concept of integrated maternity care described mentioning attributes, boundaries and consequences.’ In this concept analysis we systematically analyzed the different definitions and use of the concept integrated maternity care. Analyzing the literature, using Morse’s methodology on concept analysis, led to the identification of three defining attributes on integrated maternity care: integrated maternity care is about 1) collaboration, 2) organizing the collaboration, and 3) woman-centeredness. Based on the analysis of an extensive number of records we suggest the following definition: ‘Integrated maternity care is woman-centered care provided by (maternity) care professionals collaborating together within and across different levels of healthcare with a specific focus on organizing seamless care.’ The intention of integrated maternity care is to achieve continuity and efficiency of care, and improved outcomes for mothers/families and their children.

Collaboration was the only attribute mentioned in all 36 records included in our study. From the data, it became clear that collaboration is about interaction within and across different levels of healthcare: systems, sectors, organizations, professionals, or a mix of these actors. When comparing literature on integrated maternity care with literature on integrated care in general, it becomes apparent that both fields advocate collaboration between various actors and levels, mentioning the same challenges in organizing the collaboration [2,3,6,7,9,11,16–21,27,29–31,33–42,44–47,49,51,52]. The complexity of organizing this collaboration is illustrated in several papers, where various frameworks and models are suggested that can be used to effectively implement integrated care [3,6,52].

Organizing this collaboration was the second attribute we identified. Organizing ranges from informal networks to partnerships to merged organizations [7,11,16,21,26,28,31,32,34,38–40]. The term network was used most in the context of organizing integrated maternity care where network types range from informal to highly organized networks. In literature on organizing networks, or network governance, we found that some form of governance seemed required, regardless of the formality of the organization [53]. As indicated, collaboration in integrated maternity care takes place at different levels of healthcare, for example between different professionals or between different organizations, each with its own goals and interests. However, there are also some shared goals accepted by all, such as improving the quality of maternity care. A multilevel perspective on collaboration is needed to understand how those individual and shared goals can be achieved. Once this is clear, it is possible to see which governance fits the network. Van der Weert et al. (2022) indicated that, for example, the professional level requires different governance than the organization level, because professionals often prioritize autonomy and the ability to practice their skills, while organizations prioritize structure and adherence to rules and regulations. Balancing these different needs can be challenging, but it is important to recognize that both are necessary for providing integrated care. This requires different governance at different levels in a network, which is a complicated process to understand and implement [53–56]. To successfully collaborate and provide integrated maternity care, it is important that all actors collaborate consistently within and across different levels of healthcare. It is not something that can be rushed or done quickly [56]. In the literature, little attention is paid to funding and the complexity of financing the collaboration [53]. Also in our study, only five records, of which four from the Netherlands, mentioned a need to change financial structures when organizing integrated maternity care [9,30,38,40,42]. This financial change was also identified as a bottleneck in implementing integrated maternity care and needs further studies to identify factors and strategies for successful implementation [13,56].

We identified woman-centeredness as the third attribute of integrated maternity care. We did not find a clear description of woman-centered care in integrated maternity care. Overall, it is about meeting women’s needs and coordinating care within and across different levels of healthcare that are directly involved in the care for women [3,57]. The concept of woman-centeredness seems strongly shaped by interpretations and perspectives and remains implicit, which is a barrier for practice [3,4,15]. To further implement woman-centered integrated maternity care it is necessary to define the concept of woman-centeredness with the collaborating actors in integrated maternity care.

One of the antecedents we identified for integrated maternity care is “role clarity”. To provide integrated maternity care, it is important that all involved professionals are aware of each other’s competencies, tasks and responsibilities. We described two different views on responsibilities. Most records mentioned there should be equal or shared responsibilities [11,18,35,44,48]. Only Perdok et al (2016) suggested a strict distinction between tasks and autonomous responsibility of the involved professionals should be pursued [42]. In our included records, it was not clearly described what responsibility means or how this could be organized. Heatley et al (2011) described this division of responsibilities in interprofessional collaboration in maternity care as each care provider is responsible and accountable for their own actions but the outcome should be a joint responsibility [54]. This division is also acknowledged in literature on integrated care in general [2,3,57]. Unlike what we found in our study, literature on integrated care in general explicitly mentioned accountability as an important attribute of integrated care [3,57]. Accountability should be considered when organizing integrated maternity care. Collaboration within and across different levels of healthcare, and especially working in networks, is challenging in health care. For example, there may be inequality between actors involved, where some contribute fewer resources than others or others are larger. But there are also frequent differences in views, or differences in knowledge and skills. This makes equal discussions between different disciplines and across organization boundaries complex [58]. Within the context of Dutch maternity care, we observe that the development of integrated maternity care is strongly influenced by these differences [13]. In order to promote further collaboration in a network, Van der Scheer (2022) has emphasized the necessity of finding a solution that honours the differences between involved actors while maintaining their equivalence [58]. She mentioned three options that could contribute to finding this solution namely creating formal arrangements or working collaboratively through a combination of trust and informality or a combination of both [58]. It is not yet clear which of these options will work best to further implement integrated maternity care in the Netherlands. The antecedent we identified: ‘the presence of a culture of collaboration” links to building mutual trust between actors in order to provide integrated maternity care. Goodwin (2016) also described mutual trust as an important prerequisite to integrated care in general needed to facilitate collaboration between all actors [2]. Next to mutual trust, respect, and understanding each other’s approach to practice are important prerequisites to integrated care [2,3,54].

In the Netherlands, integrated maternity care started with the registration of the Integrated Maternity Care Standard in 2016 [7]. The main focus of this standard is how to organize woman-centered maternity care to improve quality of care. This ‘how’ is focused on organizing collaboration between professionals. It does not address the antecedents we found in our study. This focus on organization is also seen in literature on implementing integrated care in general. Goodwin (2016) described that, when implementing integrated care, the focus is often on organizational solutions, but changing care is only possible in the interface between all actors involved [2]. Thus, focussing on the antecedents of integrated care should be central in the implementation of integrated care. From our study we suggest that a focus on role clarity, creating a collaborative culture (mutual trust, respect and understanding each other’s approach), and arrange timely and clear communication should be prior to organizing the collaboration in formal structures. These elements are also part of the values contributing to the successful implementation of integrated care in general as mentioned in several studies [3,54,57,59,60].

The improved outcomes, including perinatal outcomes and women’s experiences, and efficiency of care, we identified in our study as consequences of integrated maternity care were mostly not based on research but on suggested models of integrated maternity care and its potential impact on the quality of maternity care [7,9,11,20,30,31,36,38,50]. Next to the different definition of outcomes, integrated maternity care was also differently interpreted in the records. Because of the use of these different interpretations, results of the different studies were not comparable, which was also concluded by de Jongh et al (2016) and Smeets (2022) [14,15] in their respective studies on integrated antenatal and primary care. This finding is also confirmed by Van der Weert et al. (2022) who concluded in their systematic review, that there is hardly any empirical research available on the consequences of integrated care [53]. Further research on consequences of integrated maternity care, requires a clear definition of integrated maternity care [15]. This concept analysis addresses this need.

### Strengths and limitations of the study

This concept analysis is the first of its kind on integrated maternity care, which adds to the existing knowledge on this important concept. Morse’s method of concept analysis ensures that the concept of integrated maternity care is clearly defined and its attributes, boundaries, antecedents, and consequences are identified in a structured way. Since no checklist is specifically designed for concept analysis, we applied the PRISMA checklist for systematic reviews. Not all items of this checklist were relevant, still, it enabled us to ensure a systematic and rigorous approach in our concept analysis methodology [61]. Incorporating both research and non-research literature in this concept analysis offers a comprehensive understanding of the concept, resulting in a more complete and accurate analysis. The present concept analysis encountered challenges in comprehensively identifying all literature pertaining to integrated maternity care within the context of organization, potentially due to the heterogeneous usage of key terms such as "shared care" or "team-based care" in the literature. Of the 36 included records, the majority (n = 15) originated from the Netherlands and described care as outlined in the Integrated Maternity Care Standard, which may introduce some bias to the findings. To minimise bias, we paid explicit attention to overrepresentation of literature from the Netherlands and consciously checked the analysis for sufficient international representation. The data utilized in this analysis comprised both research (n = 21) and non-research (n = 15) records, displaying a diverse range of content and context. The limited availability of studies with quantitative data leaves scientific underpinning of the requirements and effects of integrated maternity care open.

## Conclusion

Our proposed definition of integrated maternity care is: ‘Integrated maternity care is woman-centered care provided by (maternity) care professionals collaborating together within and across different levels of healthcare with a specific focus on organizing seamless care.’ This definition can contribute to understanding the concept of integrated maternity care. Understanding is a necessary step in implementing and evaluating integrated maternity care. Additionally, the identified antecedents are useful for the successful implementation of integrated maternity care. It seems essential to shift the focus on its governance/organization form to the conditions necessary to facilitate collaboration. Attention to informal methods, such as building trust, may play a more significant role when starting than governance. Our analysis highlights the importance of considering these factors to effectively implement and study integrated care. The definition of integrated maternity care that we have provided in this concept analysis can contribute to further research on the effects of integrated maternity care and how to organize and finance it.

## Supporting information

S1 File Data extraction table. (XLSX)
